# Deliberate Self-Harm and the Elderly: A Volatile Combination—An Overview from the Plastic Surgery Perspective

**DOI:** 10.1155/2012/740378

**Published:** 2012-06-19

**Authors:** J. Packer, M. A. Hussain, S. H. A. Shah, J. R. Srinivasan

**Affiliations:** Plastic Surgery Department, Royal Preston Hospital, Lancashire Teaching Hospitals, NHS Foundation Trust, Preston PR2 9HT, UK

## Abstract

*Aims*. To study the factors associated with the DSH in the elderly group of 60 years and above and to recommend changes to be implemented in order to improve the management in this specific group. *Materials and Methods*. Five-year retrospective study was undertaken from July 2005 to July 2010 in the Plastic Surgery Department of the Royal Preston Hospital, NHS Trust. A Performa was designed to collect data about the inpatient admission and included certain areas of key information. The case notes for all patients were extensively analysed in order to gather adequate information for the devised Performa. *Results*. DSH is getting more common in the elderly group, and males are more affected than females. 60% of the patients had a previous history of DSH. A large number (80%) of patients had a previous history of mental illness. 60% of those DSH patients were living with family. Almost all patients (90%) were reviewed by the Psychiatry Liaison Team. The timing of patients being assessed was highly variable. *Conclusions*. Marriage is not a protective factor in the prevention of the DSH in the elderly group. A mental health team referral in the early phases of the management would be of huge benefit and a likely step to prevent possible future admissions. The Department would benefit from the creation of a protocol for the management of these patients. There should be a joint effort of the professionals in the management of DSH in the elderly, and GPs play a very important role in the prevention of DSH in the later life.

## 1. Introduction

Deliberate self-harm (DSH) is defined as the direct, intentional injuring of the body without suicidal intent [1], and the resulting wounds can range from superficial cuts to a “full house,” involving tendons, nerves, and important blood vessels [2]. These self-inflicted injuries are a common source of referrals to hand trauma units and thought to account for approximately 150,000 cases per year in the UK [2]. DSH is commonest in the younger population, often first appearing between the ages of 14 and 24 [3]. While the number of elderly patients presenting with DSH is much lower than in the comparative younger age group, recent observations suggested a rise in DSH, particularly in elderly men. Also, it is thought that DSH in the elderly comes with an increased risk of serious injury and suicide [4]. It appears that, amongst this older age group, deliberate self-harm is in fact a strong predictor of subsequent suicide. According to a new case control study comparing 76 adults, the majority of elderly people who had deliberately harmed themselves had high suicide intent.

Plastic surgeons are often involved in the initial assessment and surgical management of patients presenting with DSH injuries [5], and these patients can often pose a challenge to the plastic surgery team [6]. The National Institute for Clinical Excellence guidelines in 2004 attempted to improve management and prevention of deliberate self-harm. However, despite these guidelines, it seems that there is ongoing inconsistency in the management of psychiatric patients. This is thought to be related to the differing levels of psychiatry exposure that doctors undergo as part of their medical training [5]. Plastic surgeons may act as gatekeepers to more specialist care for these patients [5]. It is important that we are aware of how to access these services quickly and effectively, to ensure that the safest discharge and management plan in place to prevent further attempts of DSH.

Another key issue is that these psychiatric patients require labour intensive care and expertise that may be better provided by psychiatric staff [2]. A recent article outlining the issues experienced at Whiston Hospital re-iterates that these patients represent a challenge for plastic surgeons nationally. The Plastic Surgery Department there subsequently developed a management algorithm in order to smoothly admit and refer patients with DSH and received good positive feedback from the staff who regularly uses it [6].

Literature review revealed a study of 145 patients attending a general hospital with self-harm, highlighting some interesting facts about DSH. Of the patients seen, 90% had depressive illness, 3% established dementia, and 63% had significant illness. Half the patients received inpatient psychiatric treatment. Fewer than 10% of the patients gave a history of earlier self-harm [7]. Marriage may no longer be a protective factor in prevention of DSH among older men [8]. GPs may have an important role to play in prevention of DSH in later life [8].

The aim of this study is to investigate the management of those elderly patients attending the Plastic Surgery Department at Royal Preston Hospital following presentation with deliberate self-harm. This study investigates any common factors associated with these patients and suggests improvements that can be made in the management of this specific group.

## 2. Materials and Methods

The study was carried out at the Plastic and Reconstructive Surgery Department of Royal Preston Hospital (RPH), Lancashire Teaching Hospitals NHS Trust. The aim of the study is outlined in Table 1 below.

The study investigated cases retrospectively over a 5-year period from July 2005 to July 2010. Ten patients over the age of 60 years were identified as being treated for the deliberate self-harm. During the study those patients who presented with DSH and satisfied the inclusion criteria over the time period were selected. See Table 2 below.

A proforma was designed to collect data about the inpatient admission and included certain areas of key information. The case notes for all patients were extensively analysed in order to gather adequate information, and Table 3 below shows an example of the proforma used.

## 3. Results

**Figure 1:**
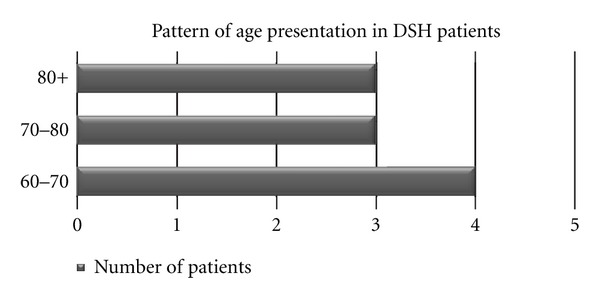
A bar chart showing the pattern of the age groups of patients presenting with DSH to Plastic Surgery Department.

There was variation in age groups presenting to the department, and this was evenly spread across the age categories (Figure 1). However, the data highlights a greater number of male patients (70%) presented with DSH than female patients (30%). This correlates with the recent data suggesting a rise in incidence among elderly men.

All of the patients presenting to the Department used knives to inflict DSH, and all of these presented with hand lacerations. It was not always documented as to whether any alcohol or drugs were taken in addition to the knife injury. In one patient, there was documentation of paracetamol overdose deemed not within alarming limits (following N Acetyl cysteine blood level measurement).

**Figure 2:**
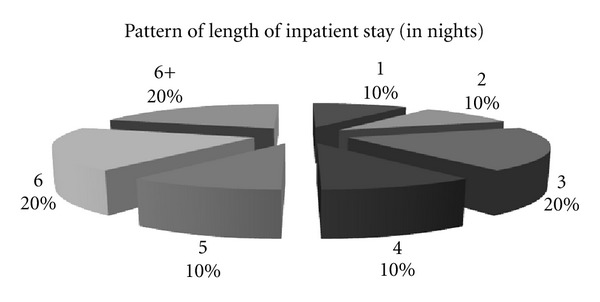
A pie chart showing the length of stay of patients in Royal Preston Hospital.

Most commonly, the patients were discharged within 6 days (80% of patients). On 2 occasions patients had extended stays in hospital. On both occasions this was due to social circumstances limiting discharge, and the involvement of the necessary services. One patient stayed for 65 nights in the hospital (Figure 2).

Half of the patients were referred to the Department from either Chorley or Preston Emergency Departments. In the other half of cases, the patients were referred from Blackburn Royal Infirmary, Burnley General Hospital, Blackpool Victoria Hospital, or Accrington Hospital. Most patients presented within the first 24 hours of injury (80%). Only 2 patients attended after 24 hours of injury.

**Figure 3:**
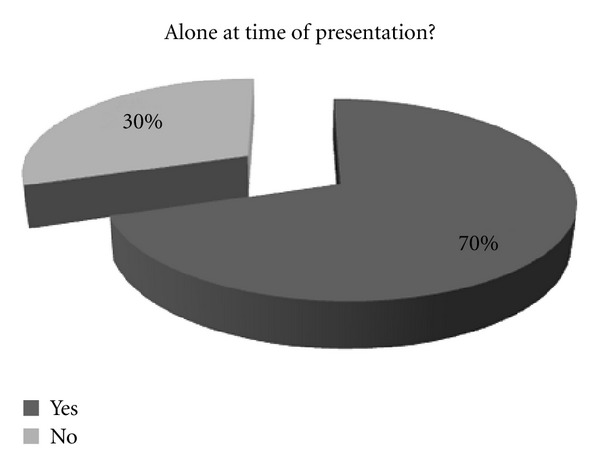
A pie chart showing the pattern of whether patients attended alone or with family.

About 70% of the patients attended the emergency department alone (Figure 3). Rest of the 30% of the patients who did not seek medical attention alone were men, and all of them attended with their wives. Of these patients, 1 did not voluntarily attend the Emergency Department. He was found collapsed by the canal with a bleeding wrist, and brought in by Police.

**Figure 4:**
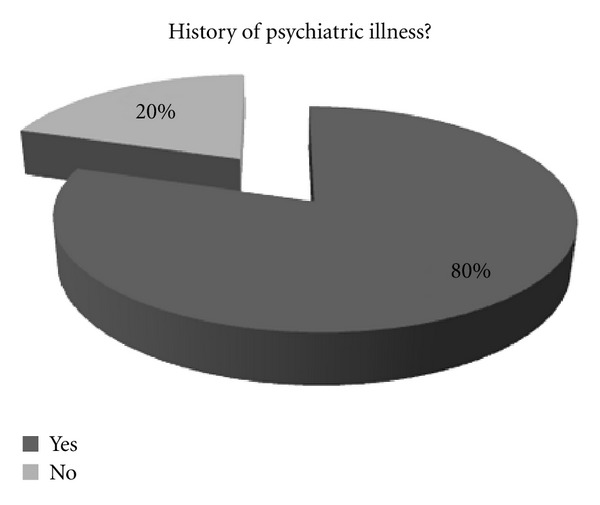
A pie chart showing the pattern of previous mental illness amongst those patients presenting with DSH.

For more than half (60%) of the patients who presented with DSH, this was not the first time. 60% of the patients had a previous history of DSH. A large number (80%) of patients had a previous history of mental illness. This highlights the chronic manifestation of mental health problems (Figure 4).

**Figure 5:**
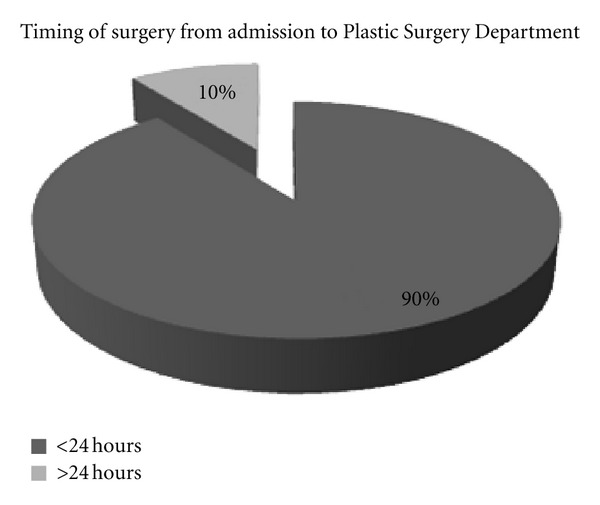
A pie chart showing the timing of surgery from admission.

Whilst living with family is documented in literature to be thought as a protective factor for mental illness, 60% of those patients who presented were living with family. Almost all patients were reviewed by the Psychiatry Liaison Team. The bar chart below highlights that the timing of patients being assessed was highly variable. 5 patients were seen after longer than 24 hours whereas 4 patients were seen within 24 hours of admission. Most patients were operated on within 24 hours of admission (90% of patients) (Figure 5).

Just over half of the patients did attend their follow-up appointments including physiotherapy, hand therapy, and outpatient clinic follow-up appointments.

## 4. Discussion

Prevalence of personality disorder was more in the elderly group, and males are more involved [9], which is clearly reflected from this study. Whilst living with family is normally thought as a protective factor for mental illness, 60% of those patients who presented were living with family. This shows that marriage is not a protective factor in the prevention of the DSH in the elderly group and that correlates to the documented evidence in the professional literature that marriage may no longer be a protective factor in prevention of DSH among older men [8].

According to a new case control study comparing 76 adults, the majority of elderly people who had deliberately harmed themselves had high suicide intent. Our study population had a psychiatry liaison assessment rate of 90%. However, the timing of the assessment was highly variable, and 50% of patients had to wait at least 24 hours before they received any psychiatry input.

It is very important that these patients should be managed using a multidisciplinary method. They should be assessed by the psychiatric team on the day of admission, and if they need admission, they should be admitted under their care and not under the care of the plastic surgery team. This way we can prevent the delay in the start of the psychiatric treatment which is more beneficial to this group of patients, as deliberate self-harm in the elderly comes with an increased risk of serious injury and suicide [4].

### 4.1. Limitations

There are several limitations to the study. Only patients coded as “deliberate self-harm” will be highlighted on the patient data search conducted by the Audit Department. This may potentially miss some patients, simply because the notes may have been coded incorrectly, depending on what had been documented in the discharge summary.

Another limitation relates to the documentation during the inpatient stay. The retrospective nature of this audit means that it relies heavily on documentation. Several of the questions on the proforma warrant information that may sometimes not be documented, such that the exact date and time certain assessments were undertaken. This is straight forward for operation dates, but not always for other assessments, for example, psychiatry liaison team review.

Also, not all patients admitted with deliberate self-harm are referred to Plastics. Some patients may be seen and treated in other specialties. Another limitation is that the Plastic Department will only be referred hand trauma, and not trauma relating to other sites.

## 5. Conclusion

DSH is getting more common in the elderly group, and males are more affected than females. In our study we found out that 60% of the patients had a previous history of DSH. A large number (80%) of patients had a previous history of mental illness, which clearly shows the chronic manifestation of mental health problems in this study population.

The results highlighted that the Department would benefit from the creation of a protocol for the management of these patients, similar to that now in use at the Plastic Surgery Department at Whiston Hospital. It would seem that referral to mental health services in the early phases of the management would be of huge benefit, and a likely step to prevent possible future admissions. Management of this specific group should involve the joint effort of the professionals which comprises of psychologist, psychiatrist, surgeons, social services, and the GP. GPs may have a very important role in the prevention of DSH in later life [8]. It is a growing burden on the nations' health care and presents a management challenge for all of us. We definitely need to work hard towards providing effective management and preventive strategies.

## Figures and Tables

**Table 1:** Aims and objectives of the study.

Aims and objectives	
(i) Investigate factors commonly associated with the DSH in the elderly. (ii) Investigate whether management of DSH in elderly differs to the management of DSH in younger population? (iii) Discover how management of these patients can be improved. (iv) Discover what professionals can do to help this subgroup.	

**Table 2:** Inclusion criteria for the study population.

Inclusion criteria
(i) Age over 60 years (ii) Presentation over past 5 years (iii) Presentation to Plastic Surgery Department at RPH	

**Table 3:** Patient data collection proforma.

Study proforma; information-gathering tool
(i) Age (ii) Gender (iii) Length of inpatient stay (number of nights) (iv) Pattern of referral to the Department (v) Timing of presentation to medical services following injury (vi) Mode of injury (vii) Was patient alone at the time of presentation? (viii) History of previous DSH (ix) History of previous mental illness (x) Was the patient living with family or alone? (xi) Inpatient psychiatry liaison assessment done? (and time of this) (xii) Time to surgery from presentation (xiii) Postoperative plan differed from routine plans? (xiv) Did patient attend follow-up outpatient appointments?	
